# General characteristics and reasons for the discontinuation of drug clinical trials in mainland China

**DOI:** 10.1186/s12874-021-01443-2

**Published:** 2021-11-13

**Authors:** Ben-nian Huo, Mao-lin Ai, Yun-tao Jia, Yao Liu, Yang Wang, Nan-ge Yin, Lin Song

**Affiliations:** 1 Department of Pharmacy Children’ s Hospital of Chongqing Medical University, National Clinical Research Center for Child Health and Disorders, Ministry of Education Key Laboratory of Child Development and Disorders, Chongqing Key Laboratory of Pediatrics, Chongqing Clinical pharmacy Key Specialty Construction Project, Chongqing, China; 2 Department of Pharmacy, Daping Hospital, Army Medical University, Chongqing, China

**Keywords:** Clinical trials, Discontinuation, Drug trial registration and information publication platform, China

## Abstract

**Background:**

Although discontinuation is common in clinical trials, no study has been conducted to analyse the current situation and reasons for the suspension or discontinuation of drug clinical trials in China. This study aims to analyse the general characteristics and reasons for the discontinuation of registered clinical trials in mainland China and to identify the associated factors.

**Methods:**

We conducted a cross-sectional observational study of discontinued trials registered in the Drug Trial Registration and Information Publication Platform before March 31, 2020. All trials with a status of terminated or stopped recorded in the platform were classified as discontinued trials and included in the analysis. The basic characteristics of the discontinued trials were recorded, reasons for trial discontinuation were recorded and divided into 4 categories as drug development strategy, trial planning, trial conduct and studied drug. Pearson’s chi-square test and fisher’s exact test were used to compare the differences in reasons for discontinuation between neoplasm trials and non-neoplasm trials, and to examine the associations of trial characteristics with different reasons related to trials discontinuation.

**Results:**

Three hundred twelve discontinued trials were included in this study. The studied drugs were mainly chemical drugs [229 (73.4%)], and indications of the studied drugs were mainly neoplasms [77 (24.7%)]. Geographical location of the discontinued trials were mostly in northern [114 (36.5%)] and eastern [96 (30.8%)] China. Study type of the included trials was mainly bioequivalence studies [97 (31.1%)]. The most common reason for trial discontinuation was commercial or strategic decision [84 (26.9%)], followed by futility/lack of efficacy [70 (22.4%)]. The number of trial centers, sample size and whether participants had been enrolled were significantly associated with trial discontinuation (*P* <  0.05). Multiple center trials showed a higher rate of trial discontinuation due to trial conduct related reasons than single center trials (*P* <  0.05), trials with sample size > 500 showed a higher rate of trial discontinuation due to studied drug related reasons (*P* < 0.05), and trials enrolled participants showed a lower rate of trial discontinuation due to commercial or strategic decision and a higher rate of trial discontinuation due to studied drug related reasons than trials without enrolled participants (*P <* 0.05). Besides, neoplasm trials showed a higher rate of trial discontinuation due to poor recruitment and safety comparing with non-neoplasm trials (*P <* 0.05).

**Conclusions:**

Trial discontinuation in China mainly occurred because of commercial or strategic decision and futility/lack of efficacy of the studied drug. Clinical trials with multiple centers and a large sample size may more likely be discontinued due to trial conduct related reasons such as good clinical practice. Discontinuation due to drug safety and lack of efficacy in multiple center trials with a large sample size deserves more attention to avoid resources wastes. Full communication with regulatory authorities such as Center for Drug Evaluation and research institutes to develop a feasible protocol is important for sponsors to avoid trial discontinuation due to protocol issues.

## Background

From 2012 to 2014, there was a large backlog of New Drug Applications (NDA) in China, mainly because of the prolonged review time at the China Food and Drug Administration, the application time of new drug was extended from 4 to 9 months and the queuing time of NDA from 12 to 15 months [1]. In 2014, the pending drug registration applications increased to 18,597,[2] the impact of the drug approval lag became a huge obstacle to the pharmaceutical industry in China. However, since 2015, a series of measures have been taken to promote drug development, speed up the review and approval process, and transform it from strict entry and tolerant exit (i.e., it was difficult to obtain approval for clinical trials but easy to obtain marketing authorization) to tolerant entry and strict exi t[3–5]. In 2018, the approval of clinical trials changed from the so-called “nodding system” to the “shaking system”. If the applicant does not receive any negative or doubtful opinions from the Center for Drug Evaluation (CDE) of the National Medical Products Administration (NMPA) within 60 days of application acceptance, the drug clinical trials can be carried out according to the submitted protocol,[6] it significantly reduced the time for clinical trial approval, but pharmaceutical companies should be more cautious in conducting clinical trials, especially issues related to participants safety and good clinical practice, to avoid the risk of trials being halted by regulatory authoritie s[7].

Although discontinuation is common in clinical trials, stopping a trial halfway due to uncontrollable risks or any other reasons will have a considerable impact on the companies. It was estimated that more than $240 billion was wasted on discontinued clinical trials in the world every yea r[8]. Recruitment might be a major cause of discontinuation, in Switzerland, 26% of randomized controlled trials (RCTs) were discontinued due to slow recruitmen t[9]. Studies based on the RCTs registered in ClinicalTrials.gov showed that approximately 32% of trial discontinuations were due to patient recruitment issues,[10] and recruitment problems were the most common cause of trial discontinuation in pediatric RCT s[11]. Trial discontinuation may lead patients to receive unnecessary treatment interventions, cause ethical controversy, and waste financial resource s[11–14]. Additionally, discontinued trials were more likely to remain unpublished than completed trial s[15, 16]. Despite these serious concerns, few studies have examined the problem of clinical trial discontinuation, and no study has analyzed the current situation and reasons for suspension or discontinuation of drug clinical trials in China. Considering the improvement of drug supervision and management policies in recent years, it is important to analyse the characteristics and reasons for trial discontinuation in China and provide a basis for consideration of related countermeasures and reducing resource waste.

In 2012, the CDE established a Drug Trial Registration and Information Publication Platform, which is a national authoritative database for clinical trials in Chin a[17]. All drug clinical trials being conducted as registration trials including phase I–IV drug trials and bioequivalence studies must be registered on the platform before enrolment of the first patient, and the NMPA is responsible for the validity and integrity of the data,[15] this will be useful to increase transparency of clinical trial result s[16, 18]. Publicly accessible information in the platform includes trial status, sponsor, study design, and study institutions. For clinical trials stopped or terminated due to different reasons, trial status and the corresponding reason is recorded. Thus, we analyzed the general characteristics and reasons for all the discontinued clinical trials registered on the platform to provide an up-to-date and comprehensive profile of clinical trial discontinuation in mainland China and to identify the causes and associated factors.

## Methods

### Data source

We conducted a cross-sectional observational study of discontinued trials registered in the Drug Trial Registration and Information Publication Platfor m[17]. All clinical trials were identified and classified by “trial status” recorded in the database, including “Ongoing”, “Completed”, “Terminated”, and “Stopped”. As defined by CDE, terminated trials are trials that have been halted by the sponsor for different reasons (for example, negative results, security issues, lack of funding) but that may start again, and stopped trials are trials that have been halted by the drug administrative department but it will no longer start again. In this study, we classified the terminated trials and stopped studies as discontinued trials.

### Trial screening and data extraction

Trials registered on the Drug Trial Registration and Information Publication Platform through March 31, 2020 were screened for inclusion, all terminated trials and stopped trials were included as discontinued trials, ongoing trials and completed trials were excluded, and trials with registration errors which had been recorded in the database were excluded. Two authors manually reviewed the full information of all the included trials and extracted study characteristics, and further manually searched whether the included trials were restarted through the name of the drug and the sponsor, any disagreements were resolved through discussion or by consulting the third author. The following trial information were recorded:

1. Information related to researchers: the first affiliation and geographical location of the principal investigator (north, east, south, central, northeast, northwest, or southwest). The first affiliations of the principal investigators were classified into tertiary hospital, secondary hospital, scientific research institution and others. In China, tertiary hospital is a regional medical institution that provides comprehensive and specialist health services, and secondary hospital is a local medical institution that only provides comprehensive health service s[19].
2. Information related to the studied drugs: type of drugs (chemical drugs, traditional Chinese drugs, biological drugs and others) and indications. In this study, the indications of the studied drugs were coded according to the International Statistic Classification of Diseases and Related Health Problems, Tenth Revision, International Classification of Diseases (ICD)-10 classificatio n[20].
3. Information related to the trials: study phase, date of first ethical approval, date of discontinuation, study design, randomization status, blinding status, single center or multiple center trial design, control types, sample size, whether participants had been enrolled, whether a data monitoring committee (DMC) had been established, whether insurance had been purchased for participants. In this study, we recorded the date of the first ethical review as the time of the trial. Besides, DMC is also called Data Safety Monitoring Board (DSMB) or Independent Data Monitoring Committee (IDMC) in China, with the same composition, functioning, and operation modalit y[21].
4. Reasons for trial discontinuation recorded in the database were extracted and divided into 4 categories:

i: Drug development strategy including commercial or strategic decision (sponsor changed business strategy or research strategy, reform of enterprises, etc.), lack of funding and trial exemption (approved exemption by regulatory authorities, exempted due to policy update, etc.).

ii: Trial planning including protocol issues (study design issues whose impact was that the trial could not be continued or that the protocol had to be modified), poor recruitment (impractical inclusion and exclusion criteria, rare diseases with a low incidence in the general population, etc.), inadequate supplies (short supplied or expired studied drug, etc.) and study center availability and experimental conditions failed to meet requirements (laboratory could not meet the requirements or need to be redecorated).

iii: Trial conduct including good laboratory or clinical practice (self-inspection found violation of relevant regulations, etc.), poor compliance of volunteers (subjects refused to follow-up or take study drug, etc.), insufficient preparation (unable to complete the planned sample testing, etc.) and poor management ability of the principal investigator.

iv: Studied drug including futility/lack of efficacy (futility or unsatisfactory interim results) and safety (drug-related serious adverse event, toxicity).

If a single trial was discontinued with more than one reason, we extracted all the reasons recorded in the database and coded them multiple times.

### Statistical analysis

Descriptive analyses were used to summarize the data, and the number (%) was used for qualitative variables. An univariate linear regression model was used to analyse the trends in the number of discontinued trials over time, the year as the dependent variable and the number of discontinued trials as the independent variable. Pearson’s chi-square test and fisher’s exact test were used to compare the differences between neoplasm trials and non-neoplasm trials in the reasons of trials discontinuation, and to examine the association of trial characteristics and the different reasons related to trial discontinuation, bonferroni correction was conducted for multiple comparisons, and *P* values of less than 0.05 were considered to be statistically significant. All statistical analyses were performed on a personal computer with the statistical package SPSS for Windows (version 22.0).

## Results

### General characteristics of discontinued trials in mainland China

**Fig. 1:**
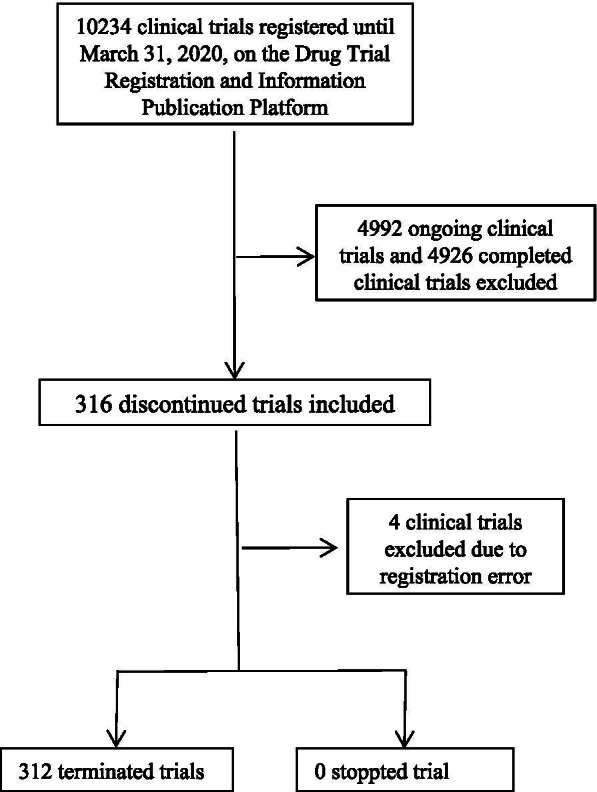
Flow chart of trial inclusion

**Fig. 2:**
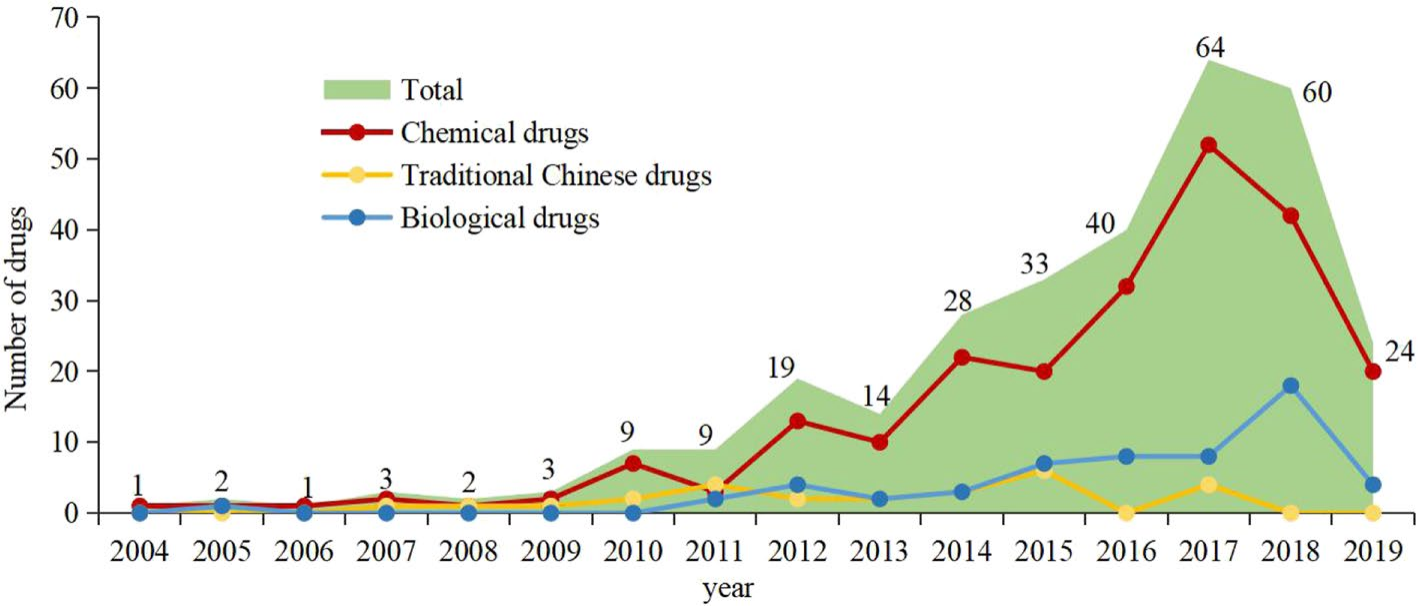
Annual number of discontinued drug clinical trials in China, 2004–2019

**Fig. 3:**
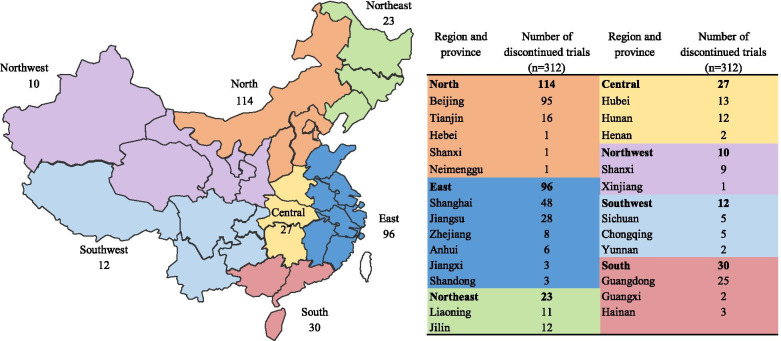
Geographical distribution of discontinued drug clinical trials in China, 2004–2019 (the map depicted in Fig. 3 was created by our engineers from the information center)

**Fig. 4:**
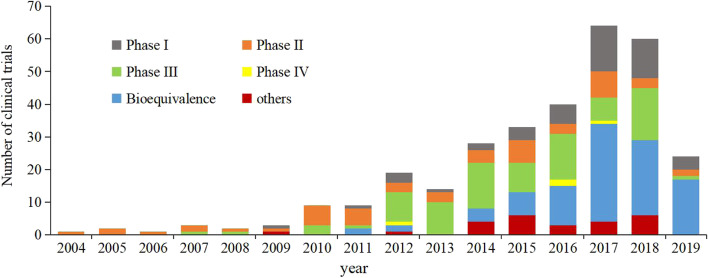
Study phase of the discontinued drug clinical trials in China, 2004–2019

A total of 10,234 drug clinical trials were registered on the Drug Trial Registration and Information Publication Platform as of March 31, 2020. Among the registered trials, 316 were recorded as terminated, no trial was stopped by the administrative department, and 4 terminated trials were excluded due to registration errors. Thus, 312 (3.0%) discontinued trials were selected for inclusion and data analysis (Fig. 1). The first ethical approval date of the trials ranged from July 19, 2004 to November 5, 2019. The annual number of discontinued trials increased significantly over time, with an average annual growth rate of 23.6% (*P*<0.001, R^2^ = 0.713). The number of discontinued trials increased obviously in 2016 and 2017, and began to gradually decrease after 2018, and a notable increase occurred in 2017 with 64 trials discontinued, corresponding to an increase of 266% relative to the number of trials initiated in 2016 (Fig. 2). The majority of the studied drugs were chemical drugs [229 (73.4%)], followed by traditional Chinese drugs [57 (18.3%)] and biological drugs [26 (8.3%)] (Fig. 2). Regarding the geographical location of the principal investigator, the discontinued trials were distributed in 24 different cities and were mostly in northern [114 (36.5%)] and eastern [96 (30.8%)] China (Fig. 3). The distribution of the indications for the studied drugs was shown in Table 1. Bioequivalence studies accounted for the largest proportion [97 (31.1%)] of the studies, followed by phaseIIItrials [86 (27.6%)], phaseIItrials [52 (16.7%)], phaseItrials [48 (15.4%)], and phase IVtrials [4 (1.3%)], the remaining 25 trials were studies on safety/efficacy and pharmacokinetics/pharmacodynamics of new drugs without a definitive phase (Fig. 4) and for trials with sample size > 500, they were mainly phase III multiple center trials (32/37, 86.5%). The general characteristics of 312 discontinued clinical trials were shown in Table 2,

**Table 1:** Distribution of studied drug indications for discontinued clinical trials in China

Indications of studied drug ^**a**^	% (n)
Neoplasms	24.7 (77)
Endocrine, nutritional and metabolic diseases	17.6 (55)
Circulatory system	9.0 (28)
Respiratory system	7.1 (22)
Infectious and parasitic diseases	5.8 (18)
Digestive system	5.8 (18)
Musculoskeletal system and connective tissue	5.4 (17)
Mental and behavioural disorders	5.1 (16)
Genitourinary system	4.8 (15)
Nervous system	4.5 (14)
Symptoms, signs and abnormal clinical and laboratory findings, not elsewhere classified	3.2 (10)
Blood, blood -forming organs,immune mechanism	2.2 (7)
External causes of morbidity and mortality	1.6 (5)
Skin and subcutaneous tissue diseases	1.3 (4)
Eye and adnexa diseases	1.3 (4)
Injury, poisoning and certain other consequences of external causes	0.6 (2)

^a^: Coded by International Classification of Diseases (ICD)-10 classification

**Table 2:** General characteristics of 312 discontinued drug clinical trials in China

Characteristic	% (n)
**Research institute**	
Tertiary hospital	91.3 (285)
Secondary hospital	1.6 (5)
Scientific research institution	1.3 (4)
Others	5.8 (18)
**Study design**	
Parallel assignment	54.2 (169)
Crossover assignment	34.9 (109)
Single group assignment	10.9 (34)
**Randomization status**	
Randomized	90.4 (282)
Non-randomized	9.6 (30)
**Blinding status**	
Open label	57.1 (178)
Single blind	4.2 (13)
Double blind	38.8 (121)
**No. of centers**	
Single center trial	45.5 (142)
Multiple center trial	54.5 (170)
**Control type**	
Active drug	49.4 (154)
Placebo control	33.7 (105)
Blank control	0.6 (2)
Uncontrolled	16.3 (51)
**Sample size**	
< 100 participants	50.0 (156)
100–499 participants	34.0 (106)
> 500 participants	11.9 (37)
Unclear	4.2 (13)
**Has enrolled participants**	
Yes	43.3 (135)
No	56.7 (177)
**DMC has been established**	
Yes	17.0 (53)
No	83.0 (259)
**Insurance has been purchased**	
Yes	48.4 (151)
No	51.6 (161)

Abbreviation: *DMC* data monitoring committee

### Reasons for trial discontinuation

Reasons for trial discontinuation were shown in Table 3. The most common reason for trial discontinuation was commercial or strategic decision [84 (26.9%)], followed by futility / lack of efficacy [70 (22.4%)], protocol issues [41 (13.1%)] and poor recruitment [35 (11.2%)]. Further manually retrieve showed 90 trials restarted, the main reason for early discontinuation of these trials was protocol issues [28 (31.1%)], it was obviously higher than its proportion in the total included trials (13.1%), and half of the trials had enrolled participants before discontinuation. In addition, 11 trials were terminated according to the DMCs’ suggestions, and the main reason for discontinuation was serious safety concerns. As neoplasm trials accounted for the largest number of discontinued trials, we further compared reasons for discontinuation between neoplasm trials and non-neoplasm trials (Table 4). Comparing with non-neoplasm trials, neoplasm trials showed a higher rate of trial discontinuation due to poor recruitment (20.8% versus 8.1%, *P* = 0.002) and safety (11.7% versus 5.1%, *P* = 0.045).

**Table 3:** Reasons for discontinuation among 312 drug clinical trials in China

Reasons ^**a**^	% (n)
**Drug development strategy**	**31.4 (98)**
Commercial or strategic decision	26.9 (84)
Lack of funding	2.2 (7)
Trial exemption	2.2 (7)
**Trial planning**	**32.1 (100)**
Protocol issues	13.1 (41)
Poor recruitment	11.2 (35)
Inadequate supplies	2.9 (9)
Study centre availability	3.5 (11)
Experimental conditions failed to meet requirements	1.3 (4)
**Trial conduct**	**8.0 (25)**
Good laboratory or clinical practice	3.2 (10)
Poor compliance of volunteers	0.6 (2)
Insufficient preparation	3.5 (11)
Poor management ability of the principal investigator	0.6 (2)
**Studied drug**	**29.2 (91)**
Futility/lack of efficacy	22.4 (70)
Safety	6.7 (21)
**No reasons given**	**1.6 (5)**

^a^Seven trials recorded a secondary reason for discontinuation, including commercial or strategic decision (*n* = 2), protocol issues (n = 2), poor recruitment (n = 1) and futility/lack of efficacy (n = 2)

**Table 4:** Comparison of the reasons for discontinuation between neoplasms and non-neoplasms trials

Reasons for trial discontinuation ^a^	Neoplasms(*n* = 77)	Non-neoplasms (*n* = 235)	*P*
**Drug development strategy**			
Commercial or strategic decision ^b^	13 (16.9)	71 (30.2)	0.022^c^
Lack of funding	1 (1.3)	6 (2.6)	1.000^d^
Trial exemption	1 (1.3)	6 (2.6)	1.000^d^
**Trial planning**			
Protocol issues ^b^	10 (13.0)	31(13.2)	0.963^c^
Poor recruitment ^b^	16 (20.8)	19 (8.1)	0.002^c^
Inadequate supplies	0 (0)	9 (3.8)	0.119^d^
Study centre availability	0 (0)	11 (4.7)	0.072^d^
Experimental conditions failed to meet requirements	1 (1.3)	3 (1.3)	1.000^d^
**Trial conduct**			
Good laboratory or clinical practice	1 (1.3)	9 (3.8)	0.460^d^
Poor compliance of volunteers	0 (0)	2 (0.9)	1.000^d^
Insufficient preparation	2 (2.6)	9 (3.8)	1.000^d^
Poor management ability of the principal investigator	0 (0)	2 (0.9)	1.000^d^
**Studied drug**			
Futility/lack of efficacy ^b^	22 (28.6)	48 (20.4)	0.137^c^
Safety	9 (11.7)	12 (5.1)	0.045^c^

Data was presented as n (%)

^a^: 5 trials did not give a reason for discontinuation

^b^: Coded multiple times for trials with multiple reasons recorded

^c^: Pearson’s Chi-square test

^d^: Fisher’s exact test

### Influencing factor analysis for trial discontinuation

Analysis of the association of trial characteristics and the different reasons related to trial discontinuation were shown in Table 5. The number of trial centers, sample size and whether participants had been enrolled were significantly associated with trial discontinuation (*P* < 0.05). Multiple center trials showed a higher rate of trial discontinuation due to trial conduct related reasons than single center trials (11.8% versus 3.5%, *P* < 0.05). Trials with a sample size > 500 showed a higher rate of trial discontinuation due to studied drug related reasons than trials with sample size < 100 and 100–499 (56.8% versus 27.4 and 21.1%, *P* < 0.05). Trials enrolled participants showed a lower rate of trial discontinuation due to commercial or strategic decision related reasons (19.1% versus 40.4%, *P* < 0.05) and a higher rate of trial discontinuation due to studied drug related reasons (46.3% versus 15.7%, *P* < 0.05) than trials without enrolled participants.

**Table 5:** Association of trial characteristics with different reasons related to trial discontinuation ^a^

Items	Drug development strategy	Trial planning	Trial conduct	Studied drug	***P***
**Study design**					
Parallel assignment (*n* = 171)^b^	57 (33.3)	58 (33.9)	9 (5.3)	47 (27.5)	0.164^d^
Crossover assignment (*n* = 111)^b^	31 (27.9)	30 (27.0)	15 (13.5)	35 (31.5)	
Single group assignment (*n* = 32)	10 (31.3)	12 (37.5)	1 (3.1)	9 (28.1)	
**Blinding status**					
Open label (*n* = 179)^b^	49 (27.4)	58 (32.4)	18 (10.1)	54 (30.2)	0.240^e^
Single blind (*n* = 14)^b^	5 (35.7)	5 (35.7)	2 (14.3)	2 (14.3)	
Double blind (*n* = 121)	44 (36.4)	37 (30.6)	5 (4.1)	35 (28.9)	
**No. of centers**					
Single center trial (*n* = 144)^b^	45 (31.3)	45 (31.3)	5 (3.5)^f^	49 (34.0)	0.028^d^
Multiple center trial (*n* = 170)	53 (31.2)	55 (32.4)	20 (11.8)	42 (24.7)	
**Sample size** ^**c**^					
< 100 participants (*n* = 157)^b^	48 (30.6)	48 (30.6)	18 (11.5)	43 (27.4)	0.002^d^
100–499 participants (*n* = 109)^b^	37 (33.9)	42 (38.5)	7 (6.4)	23 (21.1)	
> 500 participants (*n* = 37)^b^	7 (18.9)	7 (18.9)	2 (5.4)	21 (56.8)^g^	
**Has enrolled participants**					
Yes (*n* = 136)^b^	26 (19.1)^h^	37 (27.2)	10 (7.4)	63 (46.3)^h^	< 0.001^d^
No (*n* = 178)^b^	72 (40.4)	63 (35.4)	15 (8.4)	28 (15.7)	

Data was presented as n (%)

^a^: 5 trials did not give a reason for discontinuation

^b^: Coded multiple times for trials with multiple reasons recorded

^c^: We excluded trials not record planned sample size

^d^: Pearson chi-square test

^e^: Fisher’ s exact test

^f^: Significantly different from the multiple center trial group by Bonferroni correction (*P* < 0.05)

^g^: Significantly different from the < 100 participants group and the 100–499 participants group by Bonferroni correction (*P* < 0.05)

^h^: Significantly different from the no group by Bonferroni correction (*P* < 0.05)

## Discussion

In this study, we provided an up-to-date and comprehensive profile of the discontinued clinical trials in mainland China and identified the reasons and associated factors based on the national authoritative database of registration trials. On July 22, 2015, the China Food and Drug Administration issued the policy for strengthening the self-inspection and verification of clinical trial data, sponsors should do self-inspection to confirm if the clinical trial data was untrue or incomplete, and withdraw the registration application of clinical trials with data authenticity problems or other serious issues within an indicated time frame, or the new drug registration application would be rejected and the sponsors would be severely punishe d[22]. We found that about 60% bioequivalence and pharmacokinetic studies in 2016 and 2017 were discontinued due to commercial or strategic decision and protocol issues, indicating implementation of the policy effectively suppressed the existing problems in the bioequivalence and pharmacokinetic studies, and further promoted the standardization of clinical trials and reliability of the results.

There are few regulatory provisions on the discontinuation of clinical trials in China. In 2019, the CDE issued “General Risk Management and Work Procedures of Suspension and Termination in the Process of Drug Clinical Trials (Draft for Comment)”,[23] emphasizing sponsor should submit the safety update report during the clinical trial, and CDE may order to modify the protocol, suspend or terminate the trial according to the safety update report and the risk of the trial. The sponsor should submit the rectification report to CDE within 20 working days after receiving the notice of suspension or termination order. If the sponsor fails to submit the safety report, or the related serious risk or problems are not solved, the CDE may order to suspend or stop the clinical trial. Suspended trials can restart with the approval of the CDE review if the related serious risk or problems are solved. In 2020, NMPA issued “Measures for administration of drug registration”,[24] restated that the CDE may order to suspend or stop a clinical trial, depending on the risk of the trial. These measures have effectively promoted the sponsors’ emphasis on project risk management.

DMC was gradually taken seriously and established in clinical trials in China after 2019, in contrast to that only 17% included trials established DMC in our study. In 2020, the CDE issued “Guideline on Clinical Trial Data Monitoring Committees (draft)” to recommend the establishment of DMC,[21] and specified it was an independent expert group with relevant expertise to monitor the safety of subjects throughout the clinical trial, assess efficacy by reviewing the interim analysis results and assist the sponsors in making decisions such as whether to terminate the trial early due to efficacy or other problems. For confirmatory clinical trials, especially trials with large samples, high risk, complex designs, long observation periods, DMCs are necessar y[21]. With the attention of regulatory authorities, and the release of relevant policies or guidelines, more and more DMCs will be established in clinical trials in China, and then, we can further compare and analyze the value of DMCs in clinical trials including discontinued trials.

Generally, clinical trial institutions and manufacturers were mainly distributed in eastern (such as Shanghai and Jiangsu) and northern (such as Beijing) China, and most of the clinical trial institutions approved by the regulatory authorities were tertiary hospital s[25–27]. We observed the same trend for discontinued trials. Severe uneven geographical distribution of clinical trial institutions was a long-standing problem in China, it is a current challenge how to further narrow regional differences and improve the clinical trial capability in primary medical institutions.

Improving outcomes of patients with neoplasms by encouraging biopharmaceutical research and development have become a government priority all over the world, including in China, and the number of neoplasm drug trials in mainland China grew remarkably over the year s[27]. Our results showed neoplasm trials had a higher rate of trials discontinuation due to poor recruitment than non-neoplasm trials, and it was consistent with previous report s[28–30]. Poor recruitment was also a common problem in surgical clinical trial s[31] and pediatric clinical trial s[11]. Increasing the number of study centers, extending the recruitment cycle, building a flexible recruitment strategy might be valuable, but the possible additional financial burdens and the extension of the research period should be carefully considere d[32]. Previous studies showed sufficient funding and professional planning were associated with successful recruitmen t[13].

Trials with a sample size > 500, had enrolled participants showed a higher rate of trial discontinuation due to studied drug related reasons. It’s not hard to understand the safety and effectiveness of the drug can be evaluated only after the participants are enrolled and the studied drug administrated. A multiple center trial with a large sample size requires more money, manpower and other resources, and adequate early studies can effectively avoid resources waste, safety issues and ineffective results.

Multiple center trials also showed a higher rate of trial discontinuation due to trial conduct related reasons than single center trials, and mainly for good laboratory or clinical practice, insufficient preparation related reasons. Center effect such as different subject characteristics, clinical practice and management requirements can lead to heterogeneous research results and is common in multiple center trials, and severe center effect can lead to unreliable effectiveness and safety result s[33]. Sponsors should not only take into account the good clinical practice capacity of the major research center but also the sub-centers. Sufficient preparation such as a consistent management system and the standard operating procedure is important and also adequate time and ability of the researchers. Electronic management systems such as clinical trial management systems and electronic data capture systems are helpful in multiple center trials, making information exchange more convenient in the implementation stage among different center s[34].

Finally, suspension and restarting a trial due to protocol issues is also a waste of time and money, especially when subjects are already enrolled, but on the other hand, termination of a clinical trial with quality issues in time is also necessary. Although the approval of clinical trials changed from the so-called “nodding system” to the “shaking system” in 2018,[6] full consideration of the feasibility of the clinical trial protocol and detailed standard operating procedures, and also full communication with CDE and other regulatory authorities before implementation of the protocol are important to avoid unnecessary discontinuation and waste of time.

A potential limitations of this study is that the information on non-discontinued trials was not collected. Perhaps by comparing the differences between discontinued trials and non-discontinued trials, we can find some valuable information, but based on the current status of clinical trials in China, there is a high probability that the basic characteristics of the non-discontinued trials will be consistent with our findings in this study, so we didn’t collect information of the non-discontinued trials to do the comparison.

## Conclusions

Trial discontinuation in China mainly occurred because of commercial or strategic decisions and futility/lack of efficacy of the studied drug. Clinical trials with multiple centers and a large sample size may more likely be discontinued due to trial conduct related reasons such as good clinical practice. Discontinuation due to drug safety and lack of efficacy in multiple center trials with a large sample size deserves more attention to avoid resources wastes. Full communication with regulatory authorities such as Center for Drug Evaluation and research institutes to develop a feasible protocol is important for sponsors to avoid trial discontinuation due to protocol issues. Besides, narrowing regional differences further and improving the clinical trial capability in primary medical institutions is a current challenge in China.

## Data Availability

The datasets supporting the conclusions of this article are available in the Drug Trial Registration and Information Publication Platform repository, http://www.Chinadrugtrials.org.cn/index.html.

## Abbreviations

CDE: Center for Drug Evaluation
DMC: Data Monitoring Committee
NDA: New Drug Applications
NMPA: National Medical Products Administration
RCTs: Randomized Controlled Trials
